# Increased Secreted Frizzled-Related Protein 2 in Hypertension-Induced Left Ventricular Remodeling

**DOI:** 10.31083/j.rcm2505171

**Published:** 2024-05-15

**Authors:** Mengying Cao, Xueli Jiang, Xiaolin Wang, Pan Gao, Yunzeng Zou

**Affiliations:** ^1^Shanghai Institute of Cardiovascular Diseases, Zhongshan Hospital, Fudan University, 200032 Shanghai, China; ^2^Institute of Biomedical Sciences, Fudan University, 200032 Shanghai, China

**Keywords:** secreted frizzled-related protein 2, hypertension, left ventricular remodeling, cross-section study

## Abstract

**Background:**

Secreted frizzled-related protein 2 (sFRP2) is involved in various cardiovascular diseases. However, its relevance in left ventricular (LV) remodeling in patients with hypertension (HTN) is obscure.

**Methods:**

In this study, 196 patients with HTN were included, 59 with echocardiographic LV remodeling. A total of 100 healthy subjects served as normal controls. The serum-sFRP2 level was measured by enzyme-linked immunosorbent assay (ELISA). Data were collected from medical records for baseline characteristics, biochemistry tests, and echocardiography. Receiver operating characteristic (ROC) curves were used to assess the distinguishing value of sFRP2 for LV remodeling in patients with HTN. Spearman rank correlation analysis was utilized to identify factors correlated with sFRP2. Cardiac sFRP2 was determined by Western blot and quantitative polymerase chain reaction (qPCR).

**Results:**

The level of serum-sFRP2 was higher in HTN patients with echocardiographic LV remodeling than their non-remodeling counterparts. ROC analysis showed that the area under the curve (AUC) for sFRP2 in distinguishing echocardiographic LV remodeling in HTN patients was 0.791 (95% confidence interval (CI): 0.714–0.869). The sFRP2 was negatively correlated with LV dimension and positively correlated with relative wall thickness (RWT). The expression of sFRP2 was higher in hypertrophic hearts, which could be reversed by myricetin.

**Conclusions:**

The serum level and cardiac sFRP2 increased in the setting of LV remodeling and decreased by myricetin. Serum sFRP2 may be a promising distinguishing factor for LV remodeling in HTN patients.

## 1. Introduction

Hypertension (HTN) is an important and modifiable risk factor for cardiovascular diseases (CVDs), with the left ventricle (LV) being its primary target in end-organ damage [1]. The prevalence of LV remodeling in hypertensive patients was approximately 40%, which was higher than in patients with severe or refractory HTN or with a history of diabetes or CVD [2]. Sustained blood pressure (BP) overload resulted in increased LV mass index (LVMI) and relative wall thickness (RWT). According to LVMI and RWT, HTN-induced LV remodeling can be classified into three geometric patterns: concentric LVH (cLVH), eccentric LVH (eLVH), and concentric remodeling (CR) [3]. In addition to being an end-organ response, LV remodeling is an independent risk factor for adverse CVD outcomes [4, 5, 6]. CLVH possesses the highest mortality risk, and subjects with CR who reverted to normal demonstrated improved survival, whereas those who progressed to LVH had a lower survival [7]. Thus, timely diagnosis and treatment of hypertensive LV remodeling are necessary. Echocardiography is the primary tool to diagnose and evaluate LV remodeling, although its accuracy depends on the experience of the operator. Therefore, it is of important clinical value to identify any related biomarkers.

Secreted frizzled-related protein 2 (sFRP2), a vital modulator in Wnt signaling, plays an important role in cardiac remodeling caused by hypoxia [8, 9, 10], hypoxia-reoxygenation (HR) [11], pressure overload [12], and autoimmune myocarditis [13] through regulating cardiac fibrosis, hypertrophy, cell death, and regeneration. A previous animal study showed that under pressure overload, the expression of sFRP2 in the heart initially increases before decreasing alongside the development of heart failure (HF) [12]. However, there have been no clinical studies on the changes of sFRP2 in patients with HTN, meaning it is obscure whether sFRP2 is related to HTN-induced LV remodeling in humans.

Myricetin is a plant-derived flavonoid with cardioprotective effects [14, 15, 16]. Our previous study, alongside others, found that myricetin can ameliorate pressure overload-induced LVH through the BTB domain and CNC homolog 2 (BACH2)/A-kinase anchoring protein 6 (AKAP6) pathway [17] or NFE2-like bZIP transcription factor 2 (Nrf2) and JUN N-terminal kinase 1/2 (JNK1/2) signaling pathway [18]. Moreover, chronic administration of myricetin ameliorated hypertension in different animal models [19, 20]. Therefore, owing to its dual hypotensive and anti-hypertrophy effects, we hypothesized that it might affect the expression of sFRP2.

In this study, we investigated the changes in sFRP2 in the serum of hypertensive patients and in the hearts of hypertensive animals to explore whether sFRP2 can be used as an indicator of LV remodeling. We also examined the effect of myricetin on sFRP2 expression.

## 2. Materials and Methods

### 2.1 Study Design and Population 

This study adhered to the principles of the Declaration of Helsinki and was approved by the Ethics Committee of Zhongshan Hospital, Fudan University (B2020-078R). During the period of August 2020 to July 2022, a total of 325 serum samples were initially collected from HTN patients admitted to Zhongshan Hospital. Those with secondary HTN, hypertrophic cardiomyopathy (HCM), cardiac amyloidosis, valvular heart diseases, congenital heart diseases, acute myocardial Infarction (MI), HF, type 2 diabetes mellitus (T2DM), severe infection, severe renal and severe hepatic disorders were excluded from the study. Finally, a total of 196 serum samples were included for analysis. A total of 100 healthy subjects from the same period were selected as the control group (group A). Patients with HTN were divided into two groups depending on the existence of echocardiographic LV remodeling: group B (HTN without LV remodeling, normal LVMI, and RWT, n = 137) and group C (HTN with cLVH, or eLVH, or CR, LVMI ≥115 g/$m^{2}$ for men and ≥95 g/$m^{2}$ for women, or RWT >0.42, n = 59) [3]. The diagnoses of essential HTN followed the Guideline for the Prevention and Treatment of HTN in China (2018 edition) [21]. Informed consent was obtained from each participant.

### 2.2 Collection of Baseline Characteristics and Detection of sFRP2

Medical records were carefully consulted to collect baseline characteristics, including age, sex, body mass index (BMI), smoking habits, history of HTN and CVDs, medicine, biochemistry tests, and echocardiography. Echocardiography was performed by observers who were blinded to the group assignments. LVM (g) was calculated using the linear method as 0.8 × 1.04 × {[interventricular septal thickness (IVS) + LV end-diastolic dimension (LVDd) + LV posterior wall ${\mathrm{(LVPW)]}}^{3}$ – ${\mathrm{LVDd}}^{3}$} + 0.6. LVMI was calculated as LVM/body surface area (BSA). RWT was calculated as 2 × posterior wall thickness/LV end-diastolic diameter.

Fasting venous blood was centrifugated to separate serum, which was stored at –80 °C until use. A commercial enzyme-linked immunosorbent assay (ELISA) kit (YB-SFRP2-Hu, Shanghai Yu Bo Biotech Co., Ltd., Shanghai, China) was used to detect the serum-sFRP2 levels, according to the instructions. The intra-assay variation was 5.4%, and the inter-assay variation was 7.5%.

### 2.3 Animals Treatment

Male spontaneously hypertensive (SHR) and Wistar–Kyoto (WKY) rats aged 8, 12, and 20 weeks (purchased from Beijing Vitalstar Biotechnology Co., Ltd., Beijing, China) and male 8-week-old C57BL/6 mice (purchased from Shanghai JieSiJie Laboratory Animal Co., Ltd., Shanghai, China) were used in the present study. Animal experiments strictly observed the requirements of the Institutional Animal Care and Use Committee at Zhongshan Hospital, Fudan University.

The transverse aortic constriction (TAC) procedure was the same as in our previous study [17]. After surgery, mice were treated with intragastric myricetin (200 mg/kg/day) or vehicle for 4 weeks.

### 2.4 Echocardiography

Echocardiography was performed when the heart rate was approximately 400 bpm (Vevo 2100, Visual Sonics Inc, Toronto, ON, Canada). Parasternal LV long-axis M-mode images were acquired to assess cardiac function and wall thickness.

### 2.5 Quantitative Real-Time Reverse-Transcription Polymerase Chain Reaction

TRIzol (R411-01, Vazyme Biotechnology, Nanjing, Jiangsu, China) was used to extract total RNA from heart tissues. Afterward, RNA was reverse transcribed into cDNA (11141ES, YEASEN Biotechnology, Shanghai, China) for quantitative real-time polymerase chain reaction (PCR) (Q711-02, Vazyme Biotechnology), according to the manufacturer’s protocol. Gene expression data were normalized to beta-actin. The relative expression was determined using the formula $2^{-\Delta \,\Delta \,\text{Ct}}$. The primers are listed in **Supplementary Table 1**.

### 2.6 Western Blot Analysis

Proteins were extracted using radioimmunoprecipitation assay buffer (RIPA, P0013C, Beyotime Biotechnology, Nantong, Jiangsu, China) and separated by sodium dodecyl sulfate-polyacrylamide gel electrophoresis (SDS-PAGE). After blocking with 5% BSA, PVDF membranes were incubated with antibodies against sFRP2 (sc-365524, Santa Cruz Biotechnology, Dallas, TX, USA) and alpha-actinin (11313-2-AP, Proteintech, Wuhan, Hubei, China), followed by the relevant horseradish peroxidase-conjugated secondary antibody. Densitometry was performed using LAS-3000 (Fujifilm, Kanagawa, Japan).

### 2.7 Statistical Analysis

Statistical analyses were performed using R (4.0.4, https://www.R-project.org/) and GraphPad Prism (8.3.0, GraphPad Software, Inc., San Diego, CA, USA). Variables are presented as mean ± standard error, or median and interquartile range, or number and proportion. Differences were compared using the Student’s *t*-test, one-way analysis of variance (ANOVA) test, Kruskal–Wallis test along with Dunn post hoc tests, or Pearson’s chi-squared test when appropriate. Spearman rank correlation analysis was used to identify factors correlating with sFRP2. Receiver operating characteristic (ROC) curves were used to assess the distinguishing value of sFRP2 for LV remodeling in HTN patients. All statistical tests were two-sided; a *p*-value <0.05 was considered statistically significant.

## 3. Results

### 3.1 Baseline Characteristics of the Study Population

As shown in Table 1, the subjects in group A were younger, while other demographic characteristics were comparable among the three groups. The blood glucose and lipid levels were similar between the two HTN groups, although there was a significant difference when compared with group A. Group C had reduced LVDd, thickened IVS and LVPW, decreased LV ejection fraction (LVEF), and elevated cardiac troponin T (cTNT) and N-terminal pro-B-type natriuretic peptide (NT-proBNP) than group B. There was no difference in the use of antihypertensive drugs between group B and group C.

**Table 1.** **Baseline characteristics**.

	Group A (n = 100)	Group B (n = 137)	Group C (n = 59)	*p* value
Age (years)	58.2 ± 1.5	67.1 ± 0.7	62.9 ± 1.7	< *0.0001*
Sex (male%)	55 (55)	85 (62.0)	39 (66.1)	0.3368
BMI (kg/$m^{2}$)	23.6 ± 0.4	24.5 ± 0.4	25.2 ± 0.6	0.0542
Smoking (n (%))	10 (10.0)	23 (16.8)	13 (22.0)	0.111
ACEI/ARBs (n (%))	/	64 (46.7)	23 (38.9)	0.3176
CCBs (n (%))	/	66 (48.2)	29 (49.2)	0.9001
Beta-blockers (n (%))	/	50 (36.5)	23 (39.0)	0.7412
Diuretics (n (%))	/	25 (18.2)	12 (20.3)	0.7315
Hemoglobin (g/L)	134.0 (125.3, 146.5)	129.0 (117.0, 139.0)	127.0 (105.0, 143.0)	0.025
Alb (g/L)	44.0 (40.0, 47.0)	43.0 (39.0, 46.0)	41.0 (38.0, 45.0)	*0.0218*
eGFR (mL/min/1.73 $m^{2}$)	91.0 (74.0, 103.0)	79.0 (60.0, 90.0)	77.0 (44.5, 90.5)	< *0.0001*
FPG (mmol/L)	4.9 (4.6, 5.4)	4.6 (3.7, 7.5)	4.5 (3.9, 7.0)	0.7164
HbA1c (%)	5.7 (5.3, 6.3)	6.0 (5.7, 7.5)	5.9 (5.6, 7.0)	< *0.01*
TC (mmol/L)	4.21 (3.29, 5.12)	3.58 (2.77, 4.32)	3.69 (3.11, 4.56)	*0.02*
TG (mmol/L)	1.35 (0.97, 1.95)	1.60 (0.86, 2.11)	1.39 (0.91, 1.80)	0.77
LDL-C (mmol/L)	2.19 (1.53, 2.94)	1.66 (1.31, 2.32)	1.81 (1.42, 2.52)	*0.0133*
HDL-C (mmol/L)	1.20 (0.94, 1.43)	1.02 (0.84, 1.20)	1.09 (0.83, 1.28)	*0.0166*
hs-CRP (mg/L)	1.2 (0.55, 3.15)	3.3 (1.45, 13.15)	2.3 (0.93, 10.45)	< *0.0001*
cTNT (ng/mL)	0.008 (0.005, 0.026)	0.011 (0.008, 0.019)	0.027 (0.007, 0.118)	< *0.001*
NT-proBNP (pg/mL)	102.5 (40.0, 582.0)	92.1 (43.3, 358.2)	515.0 (76.3, 1720.0)	< *0.001*
CK-MB (U/L)	14.85 (12, 19.25)	15 (13, 18)	16.5 (13, 22)	0.46
CK-MM (U/L)	54 (34.5, 95.5)	58 (38.5, 85.5)	66.5 (39.5, 135.3)	0.5241
LVEF (%)	64 (61, 67)	65 (61, 67)	62 (55, 66)	< *0.001*
ARD (mm)	32 (30, 34)	35 (33, 37)	35 (32, 37)	< *0.0001*
LAD (mm)	37.6 ± 0.6	40.8 ± 0.5	40.9 ± 0.7	< *0.001*
LVDd (mm)	45 (42, 49)	49 (46, 53)	46 (43, 51)	< *0.0001*
LVDs (mm)	30 (27, 32)	31 (30, 35)	30 (27, 35)	< *0.01*
IVS (mm)	9 (8, 10)	9 (9, 10)	11 (10, 13)	< *0.0001*
LVPW (mm)	9 (8, 10)	9 (9, 9)	10 (10, 11)	< *0.0001*
PAP (mmHg)	30 (29.8, 33)	32 (30, 35)	33 (30, 38)	< *0.001*

Continuous variables are presented as mean ± standard error or median (interquartile range). Categorical variables are expressed as numbers (percentages). Statistically significant values are indicated in italic. BMI, body mass index; ACEI/ARB, angiotensin-converting enzyme inhibitors or angiotensin II receptor blockers; CCB, calcium channel blocker; Alb, albumin; eGFR, estimated glomerular filtration rate; FPG, fasting plasma glucose; HbA1c, glycated hemoglobin; TC, total cholesterol; TG, triglyceride; LDL-C, low-density lipoprotein cholesterol; HDL-C, high-density lipoprotein cholesterol; hs-CRP, high-sensitivity C-reactive protein; cTNT, cardiac troponin T; NT-proBNP, N-terminal pro-B-type natriuretic peptide; CK-MB, creatine kinase muscle-brain fraction; CK-MM, creatine kinase muscle-muscle fraction; LV, left ventricular; LVEF, LV ejection fraction; ARD, aortic root dimension; LAD, left atrial dimension; LVDd, LV end-diastolic dimension; LVDs, LV end-systolic dimension; IVS, interventricular septal thickness; LVPW, LV posterior wall; PAP, pulmonary artery pressure.

### 3.2 Serum-sFRP2 Level in Different Groups and ROC Analysis

**Fig. 1.**
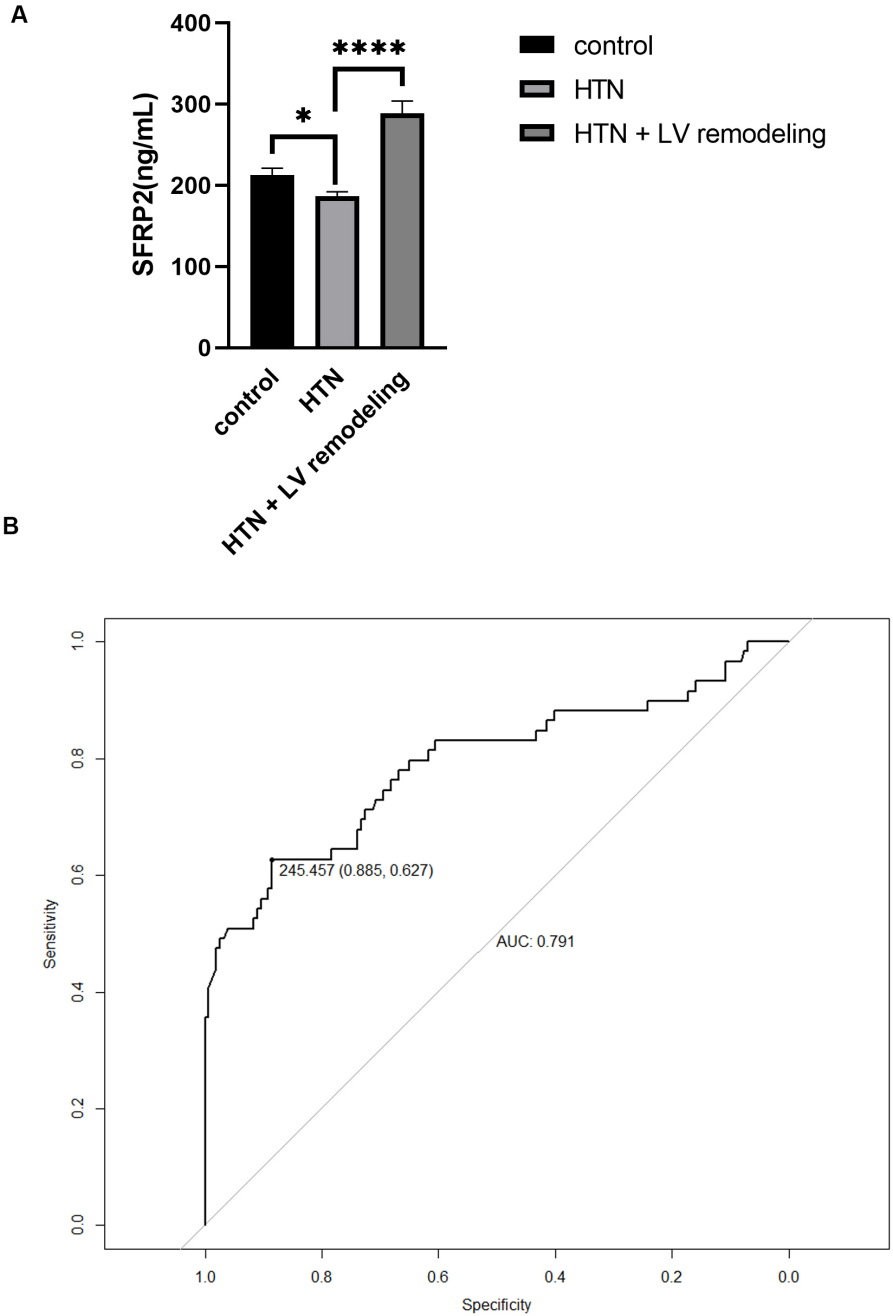
**Serum-sFRP2 levels in the different groups and ROC analysis**. (A) Serum-sFRP2 levels in the different groups. (B) The ROC curve for sFRP2 in distinguishing echocardiographic LV remodeling in HTN patients. sFRP2, secreted frizzled-related protein 2; ROC, receiver operating characteristic; HTN, hypertension; AUC, area under the curve; LV, left ventricle. *p < 0.05, ****p < 0.0001.

The serum sFRP2 in group B was lower than in group A (*p* = 0.0371) and group C (*p* < 0.0001) (Fig. 1A). ROC analysis using data from group B and group C showed that the area under curve (AUC) was 0.791 (95% confidence interval (CI): 0.714–0.869) and the optimal cutoff point was 245.475, with 62.7% sensitivity and 88.5% specificity (Fig. 1B).

### 3.3 Association between sFRP2 and Cardiac Indicators

The correlation between sFRP2 and cardiac biochemistry indicators and echocardiographic parameters was analyzed by Spearman rank correlation analysis using data from group B and group C. The sFRP2 was negatively correlated with the LVDd and LV end-systolic dimension (LVDs) and positively correlated with RWT. The detailed data are shown in Table 2.

**Table 2.** **Spearman’s correlation between sFRP2 and cardiac indicators**.

Indicator	Spearman’s rank correlation rho	*p*-value
cTNT	–0.1286671	0.07303
NT-proBNP	0.05409002	0.4634
LVEF	–0.003237633	0.97
ARD	–0.06134053	0.5168
LAD	–0.1612246	0.08659
LVDd	–0.208502	*0.026*
LVDs	–0.1943511	*0.03826*
IVS	0.1573067	0.09463
LVPW	0.1365611	0.1474
RWT	0.2322382	*0.0129*
LVMI	0.0566092	0.5497

sFRP2, secreted frizzled-related protein 2; cTNT, cardiac troponin T; NT-proBNP, N-terminal pro-B-type natriuretic peptide; LV, left ventricular; LVEF, LV ejection fraction; ARD, aortic root dimension; LAD, left atrial dimension; LVDd, LV end-diastolic dimension; LVDs, LV end-systolic dimension; IVS, interventricular septal thickness; LVPW, LV posterior wall; RWT, relative wall thickness; LVMI, LV mass index. Statistically significant values are indicated in italic.

### 3.4 sFRP2 Expression in Hypertrophic Hearts and the Effect of Myricetin

**Fig. 2.**
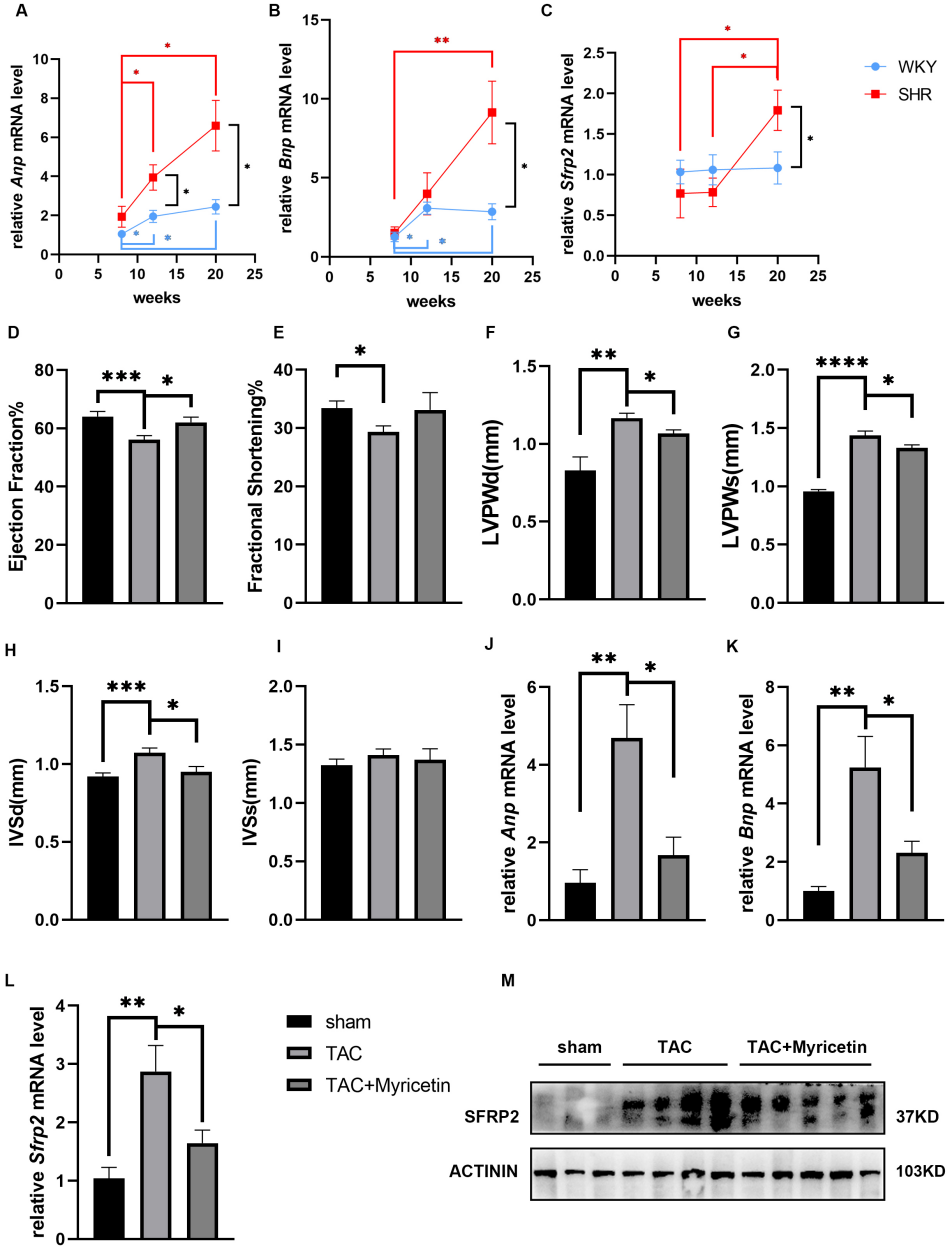
**sFRP2 expression levels in hypertrophic hearts and the effect of myricetin**. (A–C) Relative mRNA expressions of *Anp*, *Bnp*, and *Sfrp2* in the hearts of SHRs and WKY rats at different ages. SHR, spontaneously hypertensive; WKY, Wistar–Kyoto. (D–I) Echocardiography for TAC mice. LV, left ventricular; Anp, atrial natriuretic peptide; Bnp, brain natriuretic peptide; LVPWd, end-diastolic LV posterior wall; LVPWs, end-systolic LV posterior wall; IVSd, end-diastolic interventricular septal thickness; IVSs, end-systolic interventricular septal thickness; TAC, transverse aortic constriction; Sfrp2, secreted frizzled-related protein 2. (J,K) Relative mRNA levels of *Anp* and *Bnp* in the hearts of TAC mice. (L,M) Gene and protein expression levels of sFRP2 in the hearts of TAC mice, respectively. *p < 0.05, **p < 0.01, ***p < 0.001, ****p < 0.0001.

In SHRs, elevated expressions of atrial natriuretic peptide (*Anp*) and brain natriuretic peptide (*Bnp*) started at 12 weeks (Fig. 2A,B), which correlated to previous studies [22]. Although remaining nearly unchanged in WKY rats, the expression of *Sfrp2* increased with age in SHRs and was significantly higher than in WKY rats at 20 weeks (Fig. 2C). In the TAC mice, pressure overload led to cardiac function deterioration, pathological hypertrophy, and increased sFRP2 gene and protein levels, which were attenuated by myricetin (Fig. 2D–M).

## 4. Discussion

In our study, the serum-sFRP2 level was higher in hypertensive patients also possessing echocardiographic LV remodeling than in those without. Serum-sFRP2 was negatively correlated with LVDd and LVDs and positively correlated with RWT. This is the first clinical study to analyze the changes in sFRP2 levels in patients with HTN. Our previous study found that serum-sFRP2 progressively decreased when cardiac function deteriorated [23], which made us wonder how sFRP2 levels change during compensated LV remodeling. Since animal research found an initial increase in cardiac sFRP2 under pressure overload [12], we chose to detect serum sFRP2 in hypertensive patients and found a similar trend. We further detect cardiac sFRP2 in different animal models. The spontaneously hypertensive strain of WKY rat is a commonly used experimental model of hypertension since it shares many similarities to humans [24]. We used SHRs aged 8, 12, and 20 weeks to demonstrate the progress from pre-HTN to established HTN, which also compensated for hypertrophy [22], and found that the temporal increase in cardiac sFRP2 was similar to the morphological changes. It is worth noting that in SHRs, the level of cardiac sFRP2 did not change significantly from weeks 8 to 12, which was inconsistent with the result whereby serum-sFRP2 levels decreased more in group B than in group A. This difference may be explained by the fact that 8-week-old SHRs are in a pre-HTN state, which is also linked to cardiac remodeling [25]. Thus, the pre-HTN state may have a complicated effect on sFRP2. We also detected the levels in TAC mice and found increased cardiac sFRP2. However, cardiac function deteriorated at 4 weeks after surgery, which may lead to inconsistency between the animal study and clinical study due to the exclusion of HF patients.

Overexpression of sFRP2 attenuates cardiac hypertrophy by targeting the Wnt/β-catenin pathway [12]. Moreover, sFRP2 reduces HR-induced apoptosis by directly binding to Wnt3a [11]. After coronary artery occlusion (CAO), sFRP2 transgenic mice exhibited smaller infarct sizes owing to increased angiogenesis, which was mediated by activating transcription factor 6 (ATF6) and connective tissue growth factor (CTGF) [26]. Indeed, sFRP2 can also optimize the transplantation of bone marrow stromal cells (BMSCs) [9, 10] and enhance the differentiation of cardiac progenitor cells (CPCs) [27]. Based on these cardio-protective effects of sFRP2, we assume that the increase in serum sFRP2 in HTN-induced LV remodeling is a compensatory factor rather than a risk factor. This speculation is supported by the clinical study conducted by Yang *et al*. [28], which reported that sFRP2 was a compensatory factor against myocardial fibrosis in HF patients.

Although sFRP2 participates in various CVDs, its expression pattern is still not understood. Previous studies showed that pair box 2 (PAX2) [29] and sterol regulatory element binding protein-1 (SREBP-1) [30] transcriptionally activate sFRP2. Promoter hypermethylation led to the abrogation of sFRP2 in breast cancer [31]. Hence, further research is needed to explore the mechanism involved in increased serum-sFRP2 levels in HTN-induced LV remodeling.

ROC analysis showed an AUC of 0.791 (95% CI: 0.714–0.869), indicating the distinguishing value of sFRP2 for LV remodeling in HTN patients. Although echocardiography is an excellent tool, its linear method used to calculate LVM is oversimplified for hypertrophy with regional heterogeneity or dilated LV, and measurement errors can be exaggerated due to the cubing of the parameters [32]. The sensitivity and specificity of electrocardiograph (ECG) are low [33], while low availability and high costs limit the use of cardiac magnetic resonance imaging (MRI). Considering the results of the ROC analysis, serum sFRP2 may be a promising indicator.

As we predicted, myricetin reduced the pressure overload-induced elevation of cardiac sFRP2. We did not further explore whether myricetin directly regulates sFRP2 or indirectly affects sFRP2 by alleviating HTN and hypertrophy. Previous studies have revealed that myricetin modulates Wnt signaling [34, 35, 36], suggesting the possibility of its direct regulation. Notably, lifestyle changes [37, 38] and major anti-HTN drugs, including diuretics [39], renin-angiotensin-aldosterone system (RAAS) inhibitors [40, 41, 42], angiotensin–neprilysin inhibitors [43], calcium channel blockers (CCBs) [44], and beta-blockers [45, 46], can prevent and reverse HTN-induced LVH. This study showed no significant differences in antihypertensive drugs between groups B and C, reducing errors.

The presence of comorbidities, such as diabetes [47] and metabolic syndrome (MetS) [48], significantly contribute to LV remodeling. Serum sFRP2 has been reported to be negatively correlated with fasting plasma glucose (FPG) and glycated hemoglobin (HbA1c) [23] and positively correlated with BMI, total fat, and cholesterol [49]. In this study, the blood glucose and lipid levels were similar between groups B and C, excluding potential confounding factors.

Several limitations should be noted. First, the cross-sectional design precluded us from drawing causal conclusions and calls for further cohort studies or clinical trials. Second, although the ROC and correlation analyses provide some clues, they do not establish sFRP2 as a distinguishing factor for LV remodeling in HTN patients. The distinguishing value for sFRP2 needs to be validated in larger populations and needs to be compared with the gold standard echocardiography to determine the sensitivity, specificity, positive and negative likelihood ratio, positive and negative predictive value, and 95% confidence interval. Third, because of the limited sample, we combined patients with cLVH, eLVH, and CR in group C. However, different geometric patterns have specific characteristics and may have different effects on sFRP2, which needs further exploration.

## 5. Conclusions

The level of serum-sFRP2 was higher in hypertensive patients also possessing LV remodeling than those without. Serum sFRP2 may be a promising factor in distinguishing LV remodeling in HTN patients. Serum sFRP2 was negatively correlated with LVDd and LVDs and positively correlated with RWT. Cardiac sFRP2 increased alongside hypertrophy and decreased following treatment with myricetin.

## Data Availability

The raw data supporting the conclusions of this article will be made available by the authors, without undue reservation.

## Supplementary Material

Supplementary material associated with this article can be found, in the online version, at https://doi.org/10.31083/j.rcm2505171.
