# Supplementary material for: Increased Secreted Frizzled-Related Protein 2 in Hypertension-Induced Left Ventricular Remodeling
Source: Rev Cardiovasc Med. 2024 May 15;25(5):171. doi: 10.31083/j.rcm2505171 (PMC11267183; doi:10.31083/j.rcm2505171)
Supplement: Supplementary file 1 [file 2153-8174-25-5-171-s1.docx]

# Supplemental Material

Table 1. Primers used in qRT-PCR.

| Gene name | Sequences |
| --- | --- |
| rAnp | Forward: ATCACCAAGGGCTTCTTCCT |
|  | Reverse: TGTTGGACACCGCACTGTAT |
| rBnp | Forward: AAGATGGCACATAGTTCAAGC |
|  | Reverse: AGAAGAGCCGCAGGCAGAGT |
| rSfrp2 | Forward: CATGGGACAGAAACAGGGTGGA |
|  | Reverse: GAGGTCGCAGAGTGGAAGTGGT |
| rBeta-actin | Forward: CTGTGCCCATCTATGAGGGT |
|  | Reverse: CTCTCAGCTGTGGTGGTGAA |
| mAnp | Forward: GCTTCCAGGCCATATTGGAG |
|  | Reverse: GGGGGCATGACCTCATCTT |
| mBnp | Forward: GAGGTCACTCCTATCCTCTGG |
|  | Reverse: GCCATTTCCTCCGACTTTTCTC |
| mSfrp2 | Forward: GGCCACGAGACCATGAAGG |
|  | Reverse: GAAGAGCGAGCACAGGAACT |
| mBeta-actin | Forward: GGCTGTATTCCCCTCCATCG |
|  | Reverse: CCAGTTGGTAACAATGCCATGT |
